# Tree Migration-Rates: Narrowing the Gap between Inferred Post-Glacial Rates and Projected Rates

**DOI:** 10.1371/journal.pone.0071797

**Published:** 2013-08-26

**Authors:** Angelica Feurdean, Shonil A. Bhagwat, Katherine J. Willis, H. John B Birks, Heike Lischke, Thomas Hickler

**Affiliations:** 1 Senckenberg Research Institute and Natural History Museum and Biodiversity and Climate Research Center, Frankfurt am Main, Germany; 2 Romanian Academy “Emil Racoviţă” Institute of Speleology, Cluj Napoca, Romania; 3 Department of Geography, The Open University, Milton Keynes, United Kingdom; 4 School of Geography and the Environment, University of Oxford, Oxford, United Kingdom; 5 Long-Term Ecology Laboratory, Biodiversity Institute, Department of Zoology, University of Oxford, Oxford, United Kingdom; 6 Biodiversity Institute, Oxford Martin Institute, Department of Zoology, University of Oxford, Oxford, United Kingdom; 7 Department of Biology, University of Bergen, Bergen, Norway; 8 Environmental Change Research Centre, University College London, London, United Kingdom; 9 Dynamic Macroecology, Landscape Dynamics, Swiss Federal Institute for Forest, Snow and Landscape Research WSL, Birmensdorf, Switzerland; 10 Biodiversity and Climate Research Centre and Senckenberg Gesellschaft für Naturforschung and Goethe University, Frankfurt am Main, Germany; University of Copenhagen, Denmark

## Abstract

Faster-than-expected post-glacial migration rates of trees have puzzled ecologists for a long time. In Europe, post-glacial migration is assumed to have started from the three southern European peninsulas (southern refugia), where large areas remained free of permafrost and ice at the peak of the last glaciation. However, increasing palaeobotanical evidence for the presence of isolated tree populations in more northerly microrefugia has started to change this perception. Here we use the Northern Eurasian Plant Macrofossil Database and palaeoecological literature to show that post-glacial migration rates for trees may have been substantially lower (60–260 m yr^–1^) than those estimated by assuming migration from southern refugia only (115–550 m yr^–1^), and that early-successional trees migrated faster than mid- and late-successional trees. Post-glacial migration rates are in good agreement with those recently projected for the future with a population dynamical forest succession and dispersal model, mainly for early-successional trees and under optimal conditions. Although migration estimates presented here may be conservative because of our assumption of uniform dispersal, tree migration-rates clearly need reconsideration. We suggest that small outlier populations may be a key factor in understanding past migration rates and in predicting potential future range-shifts. The importance of outlier populations in the past may have an analogy in the future, as many tree species have been planted beyond their natural ranges, with a more beneficial microclimate than their regional surroundings. Therefore, climate-change-induced range-shifts in the future might well be influenced by such microrefugia.

## Introduction

Estimating rates of tree migration is critical for understanding how species range distributions are shaped by past expansion and contraction, and how species might respond in the future to climate and land-use changes. Plant range-shifts are primarily determined by climate, but life-history traits (rate of establishment, growth, survival, dispersal ability, etc.) are also important [1], [2]. Migration rates of European tree species in response to past climate changes have generally been estimated by assuming that these species persisted during the last glacial maximum (LGM) in southern Europe (southern refugia) with their northernmost distributions at approximately 40–45°N latitude [3], [4], [5], [6]. As a consequence, it has often been assumed that trees dispersed rapidly (100–1000 m yr^–1^) via long-distance dispersal in response to climate warming during the early post-glacial [3], [7]. The apparent mismatch between observed seed dispersal distances and estimates based on ecological and seed dispersal processes during the Holocene (post-glacial) has often been referred to as Reid's paradox of rapid plant migration [8]. Although palaeoecological, theoretical, and modelling studies have shown that long-distance dispersal could explain rapid migration rates [7], [9], [10], the possibility of such a rapid migration-capacity has been challenged [11].

A growing body of paleoecological, genetic, and climate-modelling literature, particularly from previously less-studied regions, such as eastern Europe and northern Asia, suggests a more northerly glacial survival for both early- and mid-successional tree species [12]–[28]. Genetic studies confirm the importance of advancing leading-edge populations for colonization [16], [29] but it is still unclear how widespread this phenomenon was. However, it is now increasingly acknowledged that these northerly populations might have acted as source populations for post-glacial expansion, in addition to the populations in more southerly locations. This has led to a paradigm shift from colonization via long-distance dispersal to rapid colonization via dispersal from local scattered populations [21], [26], [30]. Considering migration from northern refugia at the end of LGM implies that populations were closer to their present range-limits than estimated under the assumption of dispersal solely from the south, and therefore species migration rates are likely to have been lower than previously assumed [21].

Here we use fossil palaeoecological data to estimate post-glacial migration rates for eight tree taxa (including both shade-intolerant and shade-tolerant trees) taking into account re-population from northern refugia and thus assuming re-colonization via local scattered populations. These eight taxa have wide geographical ranges today in Europe and thus can be presumed to be relatively hardy and to have wide ecological tolerances. We compare these rates to estimates that assume re-colonization only from southern refugia (south of 40–45°N), and show how migration speeds are over-estimated by assuming southern refugia as the only population source. Finally, our post-glacial estimates are compared to maximum migration rates and those projected for the future with a process-based forest succession and dispersal model [31].

We find that post-glacial migration rates for trees may have been substantially lower than those estimated by assuming migration from southern refugia only. We suggest that small outlier populations may be a key factor in understanding past migration rates and in predicting potential future range-shifts.

## Materials and Methods

**Figure 1:**
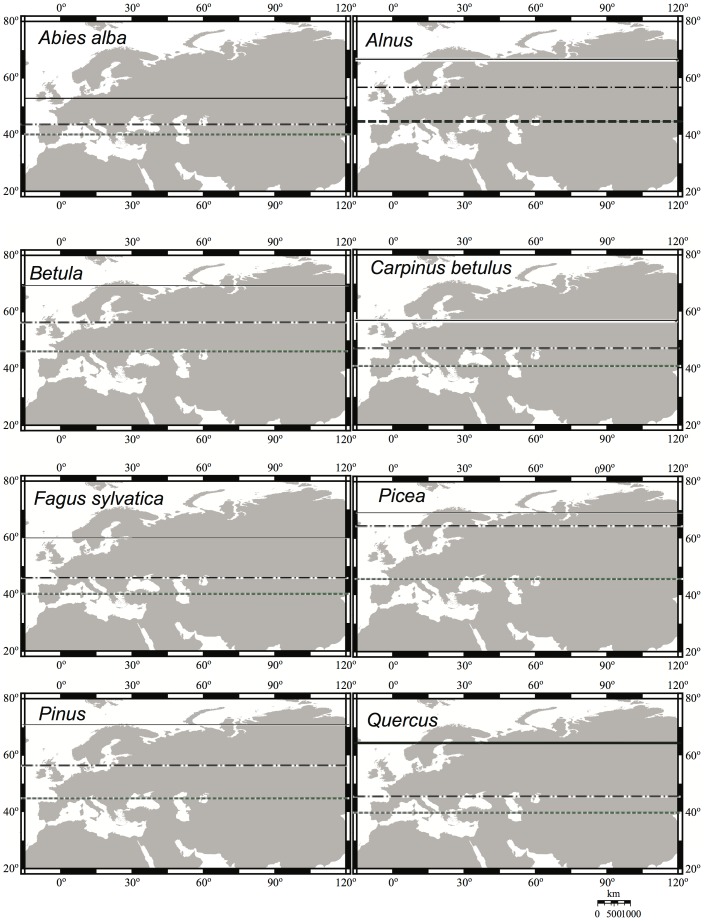
The northern range limit that a tree taxon has reached (line) either from southern refugia (dotted line) located 40–45°N or from northern refugia (dashed line) (see details in Table 1).

We determined from the Northern Eurasian Plant Macrofossil Database [24] and relevant palaeoecological literature, the northernmost geographical distributions of eight tree taxa at the end of the LGM (approximately 18,000 years ago) and the point in time when these trees reached their modern northern limits (Fig. 1; Table 1). Five out of eight tree taxa, however, are only identified to the generic level and could thus involve species with different geographical distributions, climate requirements, and dispersal modes. Compared to previous attempts at determining range shifts, we increased the taxonomic and spatial resolution by mainly using plant macrofossil remains for our migration-rate estimates. This is because pollen alone does not provide unambiguous evidence for the local presence of a species due to the problems of long-distance pollen dispersal.

**Table 1:** List of tree taxa for which fossil evidence (pollen, plant macrofossils, charcoal) exists for their survival at 18,000 cal yr BP north of 40°N.

Species	Distance from southern refugia (km) to the present day limit	Distance northern refugia (km) to the present day limit	Time of arrival at northern limit (cal yr BP)	References
*Abies alba*	1340	1100	11,500	[14]
*Alnus* (tree)	2450	1100	7000	[24]
*Betula* (tree)	2700	1300	13,000	[24]
*Carpinus betulus*	1850	1000	2000	[15]
*Fagus sylvatica*	2250	1500	1000	[16], [47]
*Picea*	2700	500	11,000	[24]
*Pinus* (tree)	2900	1550	10,000	[24]
*Quercus* (temperate)	2650	2000	6000	[5]

The distance (in km) from the perceived southern location and northern locations, respectively, and the time (calibrated years BP) when each species reached the present-day northern range limit is also given.

Cal yr BP  =  calibrated years before present (AD 1950).

**Figure 2:**
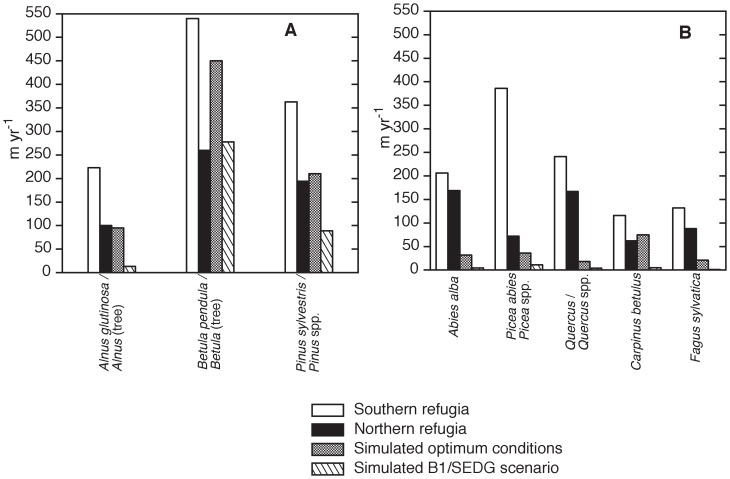
Post-glacial migration-rate estimates for (A) early-successional trees and (B) mid- to late-successional trees assuming colonization from southern and northern refugia, and comparisons with projected mean migration rates from a process-based model [from 31]. When no exact species name is available in the fossil record the genus name is used. *Picea* spp. includes *P. abies* and *P. obovata, Pinus* spp. includes *P. sylvestris* and *P. sibirica*, and *Quercus* spp. could include several species although we expect mainly temperate species such as *Q. petraea, Q. robur,* and *Q. pubescens*.

The present-day northern limits for each species were based on *Atlas Florae Europaeae*. Two sets of values were computed for each tree taxon: (1) northern migration rate, taking into account that the tree was present in northern refugia (>45°N) and spread to its post-glacial northern limit from these locations, and (2) southern migration rate, assuming that the tree only spread from southern refugia located between 40 and 45°N latitude to its post-glacial northern limit (Figs. 1, 2, Table 1). In the case of southern refugia, the maximum northern limit for cold-tolerant deciduous and coniferous tree species was estimated at 45°N, whereas for temperate deciduous trees the limit is 40°N [4], [5], [6]. The distances (in km) between the start of the migration and the northern range-limit locations were estimated linearly assuming a uniform spread from 18,000 yr BP until the time when a taxon reached its northern distribution (Figs. 1. 2, Table 1). Over-estimation (as percentages) of the migration rates was calculated as the ratio between the southern (2) and northern migration rates (1) multiplied by 100 (Table 2). In addition, we have estimated rates of migration assuming that superimposed on this long-term expansion of tree populations, tree movements could have been halted during the two major cold periods: Heinrich Event1, HE1 (lasting ∼4000 years) and the Younger Dryas, YD (∼1000 years) (Table 2)).

**Table 2:** Southern post-glacial migration-rate estimates (m yr^–1^) assuming that species spread to their present-day northern limit from the south (40–45°N latitude), and northern migration rates assuming that species spread to their present-day northern limit from their northernmost refugia.

Species	Southern fossil estimates	Northern fossil estimates	Over-estimates (fossil)	Projected mean rates (CC-Scenario B1/SEDG)	Projected max. rates (under optimal conditions)
***Early succesional***					
*Alnus glutinosa/Alnus* (tree)	225 *(410*)	100 *(185*)	225	13.6±15.3	95
*Betula pendula/Betula* (tree)	540 *(0)*	260 (0)	210	278.2±103.8	450
*Pinus sylvestris*/*Pinus* (tree)	360 *(970)*	195 *(515)*	185	89±35.4	210
***Mid- to late succesional***					
*Abies alba*	205 *(895)*	170 *(755)*	120	4.5±6.1	32
*Carpinus betulus*	115 *(170)*	60 *(90*)	185	5.0±6.5	75
*Fagus sylvatica*	130 *(190)*	90 *(125)*	150	1.1±1.7	21
*Picea abies*	385 *(1350)*	70 (*250*)	540	11.2±6.1	36
*Quercus petraea/ Quercus* spp.	220 *(380)*	165 *(285)*	130	3.6±4.5	18

Estimated rates of migration assuming no movement of taxa during the two major cold periods: Heinrich Event1 (lasting ∼4000 years) and the Younger Dryas (lasting ∼1000 years) are given in italics. Projected mean migration rates (m yr^–1^) for several tree species for 2100 using B1/SEDG greenhouse gas emission/land-use change scenarios, as well as the maximum rates derived from weak competition and optimum temperature conditions [31] are also given. When no exact species name is available in the fossil record the genus name is used.

## Results

Our analysis shows that: 1) the spreading-rate estimates from northern refugia are substantially lower than those that assume colonization only from the south, and 2) early-successional trees (*Betula, Pinus, Alnus*) migrated faster than mid- and late-successional ones (*Picea, Abies alba, Quercus*, *Carpinus betulus, Fagus sylvatica*) (Fig. 2, Table 2). The early-successional tree migration rates that assume spreading from the north vary from 100 to 260 m yr^–1^, whereas migration-rates that assume spreading from the south vary from 225 to 540 m yr^–1^ (Fig. 2A, Table 2). The mid- and late-successional tree migration rates that assume spreading from the north vary between 60 and 170 m yr^–1^ whereas migration-rates that assume spreading from the south range between 115 and 385 m yr^–1^ (Fig. 2B, Table 2). The northerly estimates are lower than those obtained by assuming survival in southern Europe only, with over-estimates ranging from 120 to 540 m yr^–1^ (Table 2). The northerly migration estimates assuming no movement of taxa during the Heinrich Event1 and the Younger Dryas range between 0 and 515 m yr^–1^ for early-successional trees and between 90 and 735 m yr^–1^ for mid- and late-successional trees (Table 2).

## Discussion

The post-glacial migration-rate estimates assuming colonization from northern populations suggest that the rates for both early- and mid- to late-successional trees are much lower than previously estimated, and that early-successional trees generally migrated faster than mid- and late-successional ones (Fig. 2). Our results also show that many taxa, in particular early- and mid-successional trees, reached their modern northern distribution in the early Holocene, a time of rapid climate changes (Table 1). The ability of early-successional pioneer taxa to persist in the harsh cold and dry LGM climate and to migrate faster than mid- and late-successional taxa would be expected as a result of differences in life history (fast growth, large seed production, far-distance dispersal) and greater stress tolerance to large amplitude temperature change, drought, etc [3], [9], [22], [32]. Generation time might also explain differences in migration rates among taxa with similar dispersal properties. For example, the early-successional trees *Pinus, Betula,* and *Alnus* first set seed at an age of 10–20 years, whereas *Picea abies* first sets seed at 30–40 years or even after 50 years [33]. Thus, although *Pinus* and *Picea* have similar seed dispersal properties, *Pinus* is able to spread much faster than *Picea* does (Fig. 2, Table 1).

Post-glacial migration rates similar to our fossil estimates have been derived from climate-driven modelled refugia at a spatial resolution of *ca* 16 km, and range between 35 and 380 m yr^–1^, with generally higher values for shade-intolerant trees (*Betula pendula, B. pubescens, Pinus sylvestris*) and for *Picea abies* than for other shade-tolerant species [2], [19]. These authors also suggest that accessibility from refugia explains to a large extent the post-glacial range shifts for many species, in particular those with a limited dispersal ability. However, they do not use fossil data as evidence of actual refugia [2], [19]. Fossil migration estimates of 250 m yr^–1^ for *Picea abies* and 100 m yr^–1^ for *Fagus sylvatica* have been recently obtained for southern Scandinavia based on pollen records and model simulation output [34] and are lower than those previously obtained of *ca.* 500 m yr^–1^ for the same region [3]. Using a similar approach as above, range displacements (contraction and expansion) between −170 and 270 m yr^–1^ have been reported from North America [35], which are higher than those previously obtained (<100 m yr^–1^) in this region based on phylogeographraphic data [29]. It is therefore evident that most species were generally only capable of migration rates less than 260 m yr^–1^. Some late-successional taxa such as *Picea*, *Abies alba*, and *Quercus* that spread quickly in the early Holocene (Fig. 2B) under conditions of low competition and low human impact should more accurately reflect their intrinsic rates of spread than other late-succession species such as *Fagus sylvatica* and *Carpinus betulus* that spread during the late Holocene and were probably dependent, to some degree, on anthropogenic disturbance of the already established forests. Our estimates indeed show fast migration rates for these species, namely *Abies alba* (170 m yr^–1^; max. 735 m yr^–1^) and *Quercus* (165 m yr^–1^; max. 285 m yr^–1^), except for *Picea* (70 m yr^–1^; max. 250 m yr^–1^), suggesting that their northern populations responded rapidly to climate change and were therefore not greatly affected by migrational lags during the post-glacial. Models of post-glacial population expansion indicate that initially tree species spread rapidly as low-density populations or isolated individuals in an advancing wave front reaching their northern limit ahead of mass colonization [34]–[37]. Later, when climate conditions became suitable and more stable, and there were higher population densities, species migrated more slowly due to increased competition from already present populations [19], [33], [35], [36]. A reduction in migration rates is also predicted to have occurred towards the species distributional limits because of less suitable climate [19], [35]–[38]. It should, however, be noted that in the case of *Picea,* for which our calculations are based on the Eurasian macrofossil data-base and thus on northern locations, migration rates are lower than the estimates for *Picea abies* [34]–[36], which are based on European pollen and macrofossil data. The migration-rate estimates for *Picea* involve two potential species, namely *Picea abies* and *P. obovata;* the latter tolerates colder temperatures and has a more northern and eastern geographical distribution than *P. abies,* which could have lowered the overall migration rates for *Picea*.

Our migration estimates are calculated assuming a uniform spread from 18,000 yr BP until the time when the taxon reached its northern distribution, despite the possibility that superimposed on this long-term expansion of tree populations, tree movements could have been halted by two major cold periods: Heinrich Event 1 and the Younger Dryas [39]–[40]. Nevertheless, our migration estimates that assume no movement during HE1 and YD (5000 years) (Table 2) change little for taxa that reached their modern northern distribution during the late Holocene (*Fagus sylvatica, Carpinus betulus*). However, our estimates under this scenario (Table 2) show high migration rates for most taxa that had already reached their modern northern distribution during the early Holocene (*Pinus, Abies, Betula, Picea*), at times of rapid climate changes. This probably reflects tree spread at low-densities in an advancing wave front ahead of mass colonization at the late-glacial/Holocene transition. Overall, we think, that assuming uniform migration rates over the entire time interval, as opposed to no movement for a period of 5000 years, appears to provide more realistic migration-rate estimates as this procedure balances fast northward movements during periods of rapid climate change and slower northward movements during periods of cold or stable climate conditions and at higher population density. Estimates of latitudinal taxa displacement from North America show dynamic changes between 16 and 12 k yr BP (expansion, contraction, stagnation), predominant fast northward expansion between 12 and 7 k yr BP and overall lower migration rates during warm and/or stable conditions occurring between 7 and 1 k yr BP [35]. Like with any fossil estimates, the spatial and temporal scales of fossil data considered for our analysis cannot provide exact locations of all northerly refugia or of the time when a taxon reached its modern northern limit. Our post-glacial migration rates should therefore be regarded as approximate estimates. Migration rates are also integrated over the varying biotic and abiotic conditions along the different migrational paths. The presence of large European mountains chains (Pyrenees, Alps, Carpathians) were previously found to not have acted as geographical barriers in the case of the spread of *Fagus sylvatica* in Europe during the last glacial–postglacial, but rather to have facilitated its survival and spread [16]. Topography might therefore be less important for many other trees considered here.

We compared our post-glacial migration-rate estimates with simulated migration rates under i) optimal conditions of competition and climate, and ii) conditions of future climate change and land-use scenarios that have been simulated using a tree migration meta-model [31]. This meta-model had been regressed against simulations of the spatio-temporal forest landscape model TreeMig [41] under various climate, competition, and fragmentation conditions and was used to constrain migration distances in an empirical species distribution model at the European scale under future land-use and resulting fragmentation and climate change. This approach thus accounts for climate influence, landscape pattern, habitat suitability, population dynamics, competition, and seed dispersal (for details see 31), unlike other models projecting the impact of 21^st^ century climate and landscape fragmentation on species range-shifts, which commonly use two extreme scenarios, i.e. unlimited or no dispersal [42], [43], [44]. The simulated rates fit better with our estimated “northern” than with the “southern” post-glacial migration rates (Fig. 2, Table 2) or with previous pollen-derived estimates [3]. The fact that the simulated rates for shade-intolerant species (mean value for future rates of 155 m yr^–1^) are of the same magnitude as those derived for the post-glacial rates implies that this is an important step towards understanding how some trees migrate in response to climate change and habitat fragmentation. Nevertheless, the simulated migration rates for shade-tolerant tree species (mean rate for the future is 15 m yr^–1^) are still an order of magnitude slower than the post-glacial migration rates, although the optimum simulated values (the maximum rate a species could move without competition or with weak competition only and without habitat fragmentation) are closer to the post-glacial estimates (Fig. 2, Table 2). This might indicate a weak influence of competition and fragmentation during the Holocene compared to the future. In addition, the reason that the simulated migration rates for shade-tolerant tree species are generally lower than the estimated post-glacial migration rates further suggests that glacial refugia for shade-tolerant tree species were farther north than currently known. Interestingly, *Picea,* whose post-glacial migration rate based on fossil data [this study, 34, 36] and hindcast model results [2], [19] suggests a rather fast migration (70–250 m yr^–1^), yields much slower rates of 10 m yr^–1^ in the future and 36 m yr^–1^ with the optimal dispersal simulations (Fig. 2). One potential reason is that, in the simulation model, *Picea abies* is assigned to the class of “medium dispersers” instead of “long-distance dispersers”, despite its (particularly at high latitudes) small seeds [45].

In summary, deriving insights on how species range distributions were shaped by expansions during the post-glacial represents a contribution to our understanding of tree-species migration rates and their likely response to future climate and land-use changes. We demonstrate that post-glacial migration estimates assuming colonization from local northern populations are much lower than previously estimated assuming colonization from the south only. Thought migration estimates presented here may be conservative because of our assumption of uniform dispersal, tree migration-rates clearly need reconsideration (Figs. 1, 2, Table 2). Although these post-glacial migration-rate estimates might change in the future when more fossil evidence becomes available, palaeobotanical data suggest that populations persisting in northern Europe during the LGM are less likely to be limited in their distribution by migrational lag. We suggest that allowing for small outlier populations is crucial for understanding past tree migration-rates and for simulating potential future changes, but this has rarely been done. The importance of outlier populations in the past may have an analogy in the future, as many tree species have been planted in small populations beyond their natural ranges, for example in parks [46], usually with a slightly more beneficial micro-climate than their surroundings. Therefore, climate-change induced range-shifts in the future might well be influenced by such microrefugia.
